# Supplementary material for: Dietary Habits in Early Pregnancy in a Multi-Ethnic Population: Results from the PROMOTE Cohort Study
Source: Nutrients. 2025 Nov 27;17(23):3729. doi: 10.3390/nu17233729 (PMC12694140; doi:10.3390/nu17233729)
Supplement: Supplementary file 1 [file nutrients-17-03729-s001.zip › nutrients-3966951-supplementary.pdf]

Supplementary table S1. Frequency histogram for daily food item intake

Vegetables:

0 – 83
1 – 145
2 – 127
3 – 37
4 – 17
5 – 6
6 – 1

25th centile – 1
75th centile – 2

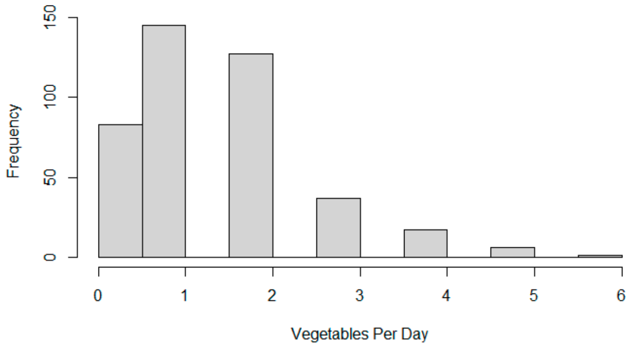

Fruit:

0 – 70
1 – 131
2 – 141
3 – 55
4 – 12
5 – 3
6 – 3
7 – 1

25th centile – 1
75th centile – 2

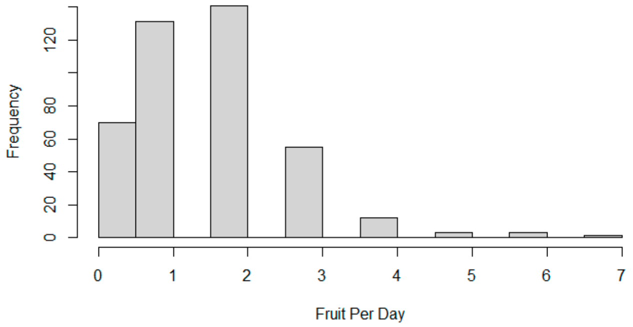

Discretionary:

0 – 12
1 – 200
2 – 126
3 – 45
4 – 15
5 – 5
6 – 8
7 – 1
8 – 2
9 – 1
23 – 1

25th centile – 1
75th centile – 2

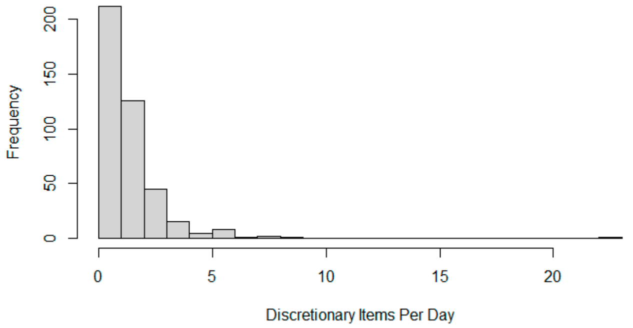

Carbohydrates:

0 – 16
1 – 71
2 – 110
3 – 81
4 – 68
5 – 23
6 – 25
7 – 15
8 – 4
10 – 1
12 – 1
17 – 1

25th centile – 2
75th centile – 4

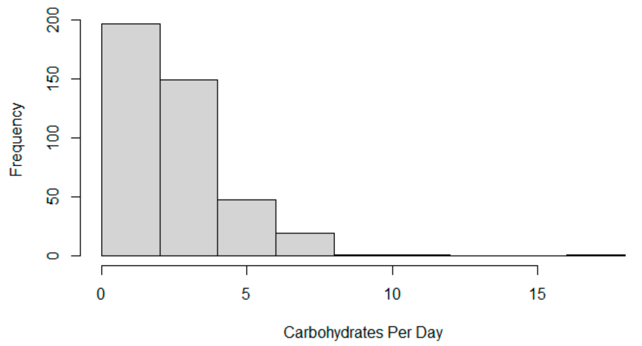

Dairy:

0 – 112
1 – 167
2 – 105
3 – 26
4 – 3
5 – 1
7 – 1
10 – 1

25th centile – 1
75th centile – 2

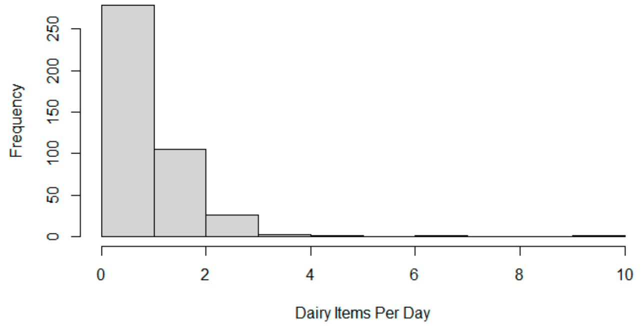

Protein:

0 – 182
1 – 202
2 – 26
3 – 6

25th centile – 1
75th centile – 2

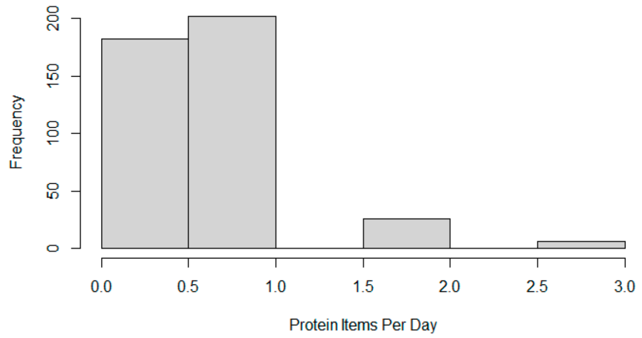

Supplementary table S2. Characteristics of those with GDM vs those without

		GDM (N = 104; 25%)	NO GDM (N = 312; 75%)	P
**BASELINE CHARACTERISTICS**	Maternal Age	34 (31 – 37)	32 (29 – 35)	0.001
	BMI	26.7 (23.6 – 31.3)	24.5 (21.8 – 28.0)	<0.001
	Any Children in Household			
	Yes	78 (76%)	211 (69%)	0.238
	No	25 (24%)	95 (31%)	
**EDUCATIONAL ATTAINMENT**	Incomplete School	3 (3%)	7 (2%)	0.762
	Complete School	7 (7%)	29 (10%)	
	School + TAFE	21 (21%)	53 (17%)	
	School + Uni	71 (70%)	214 (71%)	
**FINANCIAL STATUS**	Financial Autonomy			
	Yes	68 (66%)	229 (74%)	0.099
	No	27 (26%)	72 (23%)	
	Unknown	8 (8%)	10 (3%)	
	Household Income			
	<50K	6 (6%)	16 (5%)	0.869
	50-100K	21 (20%)	64 (21%)	
	100-200K	33 (32%)	106 (34%)	
	>200K	14 (14%)	50 (16%)	
	Unknown	29 (28%)	72 (23%)	
**MENTAL HEALTH SCREENING**				
	DASS-Anxiety			
**DASS**	Normal	62 (79%)	167 (76%)	0.336
	Mild	8 (10%)	17 (8%)	
	Moderate+	8 (10%)	37 (17%)	
	DASS-Depression			
	Normal	71 (91%)	205 (92%)	0.039
	Mild	7 (9%)	8 (4%)	
	Moderate+	0 (0%)	9 (4%)	
	DASS-Stress			
	Normal	52 (67%)	153 (69%)	0.694
	Mild	5 (6%)	18 (8%)	
	Moderate+	21 (27%)	50 (23%)	
**MENTAL HEALTH SCREENING**				
**EPDS**	Low	90 (88%)	281 (91%)	0.217
	Moderate	10 (10%)	17 (5%)	
	High	2 (2%)	12 (4%)	
**ETHNICITY**	Middle Eastern	14 (13%)	65 (21%)	0.154
	Other	6 (6%)	30 (10%)	
	S Asian	47 (45%)	106 (34%)	
	SE Asian	19 (18%)	50 (16%)	
	White	18 (17%)	61 (20%)	

**MEDICAL AND OBSTETRIC HISTORY**	Multiparous			
	Yes	81 (78%)	215 (69%)	0.104
	No	23 (22%)	97 (31%)	
	Hx GDM			
	Yes	37 (36%)	17 (5%)	<0.001
	No	67 (64%)	295 (95%)	
	Hx PCOS			
	Yes	9 (%)	26 (8%)	1.000
	No	95 (91%)	296 (92%)	
	Hx Recurrent Miscarriage			
	Yes	20 (19%)	40 (13%)	0.147
	No	84 (81%)	272 (87%)	
	Assisted Conception			
	Yes	5 (5%)	16 (5%)	1.000
	No	99 (95%)	296 (95%)	
**BREASTFEEDING HISTORY**	High Intensity BLISS			
	Yes	62 (86%)	175 (87%)	0.998
	No	10 (14%)	26 (13%)	
